# Supplementary material for: Intravesical Recurrence after Radical Nephroureterectomy in Patients with Upper Tract Urothelial Carcinoma Is Associated with Flexible Diagnostic Ureteroscopy, but Not with Rigid Diagnostic Ureteroscopy
Source: Cancers (Basel). 2022 Nov 16;14(22):5629. doi: 10.3390/cancers14225629 (PMC9688462; doi:10.3390/cancers14225629)
Supplement: Supplementary file 1 [file cancers-14-05629-s001.zip › cancers-1992629-supplementary-done.pdf]

cancers

MDPI

Supplementary Materials

Intravesical Recurrence after Radical Nephroureterectomy in Patients with Upper Tract Urothelial Carcinoma Is Associated with Flexible Diagnostic Ureteroscopy, but Not with Rigid Diagnostic Ureteroscopy

Jee Soo Ha, Jinhyung Jeon, Jong Cheol Ko, Hye Sun Lee, Juyeon Yang, Daeho Kim, June Seok Kim, Won Sik Ham, Young Deuk Choi and Kang Su Cho

Table S1. Patient demographics after PSM* in the non-URS and flexible URS groups.

	Non-URS (n=46)	Flexible URS (n=46)	*P*-Value
Age at RNU, years	68 (63–76)	69 (60–77)	0.636
Sex			0.613
Female	9 (19.6)	11 (23.9)	
Male	37 (80.4)	35 (76.1)	
HTN	23 (50)	27 (58.7)	0.402
DM	9 (19.6)	12 (26.1)	0.456
CAOD	4 (8.7)	6 (13.0)	0.503
COPD, asthma	2 (4.3)	1 (2.2)	0.557
CVA	2 (4.3)	4 (8.7)	0.398
CKD	8 (17.4)	8 (17.4)	>0.999
Smoking history			0.835
Never smoked	23 (50.0)	22 (47.8)	
Ex- or current smoker	23 (50.0)	24 (52.2)	
Tumor location			0.459
Renal pelvis	41 (89.1)	43 (93.5)	
Ureter	5 (10.9)	3 (6.5)	
Laterality			0.404
Left	24 (52.2)	20 (43.5)	
Right	22 (47.8)	26 (56.5)	
Previous/concurrent bladder cancer	11 (23.9)	8 (17.4)	0.440
Ureteroscopic biopsy	0 (0.0)	25 (54.3)	<0.001
Surgical modality			0.643
Open	14 (30.4)	12 (26.1)	
Laparoscope or Robot	32 (69.6)	34 (73.9)	
Pathologic T stage			0.785
pTa	6 (13.0)	4 (8.7)	
pT1	13 (28.3)	16 (34.8)	
pT2	7 (15.2)	5 (10.9)	
pT3-4	20 (43.5)	21 (45.7)	
Tumor grade			0.179
Low grade	6 (13.0)	11 (23.9)	
High grade	40 (87.0)	35 (76.1)	
Tumor size			0.536
<2 cm	7 (15.2)	5 (10.9)	
≥2 cm	39 (84.8)	41 (89.1)	
Lymphovascular invasion	14 (30.4)	10 (22.2)	0.374
Carcinoma in situ	5 (10.9)	2 (4.3)	0.238
Adjuvant chemotherapy	10 (21.7)	11 (23.9)	0.804
IVR within 1 year	6 (13.0)	20 (43.5)	<0.001

PSM, propensity score matching; URS, ureteroscopy; RNU, radical nephroureterectomy; HTN, hypertension; DM, diabetes mellitus; CAOD, coronary artery obstructive disease; COPD, chronic obstructive pulmonary disease; CVA, cerebrovascular accident; CKD, chronic kidney disease; IVR,

intravesical recurrence. Data are presented as *n* (%). *Estimates of PSM were calculated based on the propensity scores for age, sex, tumor location, pathological T stage, tumor grade, tumor size, and carcinoma in situ.

Table S2. Patient demographics after PSM* in the non-URS and rigid URS groups.

	**Non-URS (*n*=75)**	**Rigid URS (*n*=75)**	***P*-Value**
Age at RNU, years	67 (61–75)	68 (60–77)	0.906
Sex			0.616
Female	31 (41.3)	28 (37.3)	
Male	44 (58.7)	47 (62.7)	
HTN	39 (52.0)	44 (58.7)	0.412
DM	16 (21.3)	13 (17.3)	0.535
CAOD	3 (4.0)	10 (13.3)	0.042
COPD, asthma	4 (5.3)	10 (13.3)	0.092
CVA	3 (4.0)	5 (6.7)	0.467
CKD	14 (18.7)	5 (6.7)	0.027
Smoking history			0.247
Never smoked	47 (62.7)	40 (53.3)	
Ex- or current smoker	28 (37.3)	35 (46.7)	
Tumor location			0.166
Renal pelvis	54 (72.0)	46 (61.3)	
Ureter	21 (28.0)	29 (38.7)	
Laterality			0.412
Left	39 (52.0)	44 (58.7)	
Right	36 (48.0)	31 (41.3)	
Previous/concurrent bladder cancer	16 (21.3)	13 (17.3)	0.535
Ureteroscopic biopsy	0 (0.0)	33 (44.0)	<0.001
Surgical modality			0.453
Open	21 (28.0)	17 (22.7)	
Laparoscope or Robot	54 (72.0)	58 (77.3)	
Pathologic T stage			0.596
pTa	6 (8.0)	3 (4.0)	
pT1	27 (36.0)	29 (38.7)	
pT2	10 (13.3)	14 (18.7)	
pT3-4	32 (42.7)	29 (38.7)	
Tumor grade			0.785
Low grade	8 (10.7)	7 (9.3)	
High grade	67 (89.3)	68 (90.7)	
Tumor size			0.797
<2cm	8 (10.7)	9 (12.0)	
≥2cm	67 (89.3)	66 (88.0)	
Lymphovascular invasion	18 (24.0)	19 (25.3)	0.850
Carcinoma in situ	7 (9.3)	7 (9.3)	1.000
Adjuvant chemotherapy	20 (26.7)	21 (28.0)	0.855
IVR within 1 year	12 (16.0)	17 (22.7)	0.301

PSM, propensity score matching; URS, ureteroscopy; RNU, radical nephroureterectomy; HTN, hypertension; DM, diabetes mellitus; CAOD, coronary artery obstructive disease; COPD, chronic obstructive pulmonary disease; CVA, cerebrovascular accident; CKD, chronic kidney disease; IVR, intravesical recurrence. Data are presented as *n* (%). *Estimates of PSM were calculated based on the propensity scores for age, sex, tumor location, pathological T stage, tumor grade, tumor size, and carcinoma in situ.
